# Surgery and radiation management for chondrosarcoma of the temporo-mandibular joint: A Vietnamese case report

**DOI:** 10.1016/j.ijscr.2019.11.067

**Published:** 2019-12-11

**Authors:** Quang Van Le, Dang Van Nguyen, Hung Van Nguyen, Thanh Duc Hoang, Duy Quoc Ngo, Tung Thanh Ngo

**Affiliations:** a Department of Oncology, Hanoi Medical University, Hanoi, Viet Nam; b Department of Head and Neck Surgery, Vietnam National Cancer Hospital, Hanoi, Viet Nam; c Department of Head and Neck Radiation, Vietnam National Cancer Hospital, Hanoi, Viet Nam

**Keywords:** Chondrosarcoma, Temporo-mandibular joint, Surgery, Radiation, Vietnam, Case report

## Abstract

**Introduction:**

Chondrosarcoma is a malignant tumor originated from cartilage cells. The most common sites of chondrosarcoma are pelvis, femur and ribs. The temporo-mandibular joint (TMJ) chondrosarcoma is extremely rare, with approximately 30 cases reported in worldwide.

**Presentation of case:**

In this report, we present a case of chondrosarcoma in the left TMJ, which was successfully treated with surgery and radiation therapy. A 47 year-old women was admitted to our hospital with a mass in left pre-auricular region. On examination and para-clinical test results, the initial diagnosis was parotid gland cancer. In operation, the lesion was a mass originating from the left TMJ. Tumor resection was performed with close margin. The post-operative pathological result was well-differentiated chondrosarcoma. She was indicated adjuvant radiation therapy due to inadequate surgical therapy. There was no evidence of recurrence after 6 months follow-up.

**Conclusion:**

The TMJ chondrosarcoma is needed to distinguish from other diseases especially parotid gland tumor. Surgery and adjuvant radiation therapy are standard care with the aim of preserving joint function.

## Highlights

• The temporo-mandibular joint chondrosarcoma is extremely rare, and can be misdiagnosed as parotid gland tumor.
• Surgery is standard treatment with the aim of preserving the temporo-mandibular joint function.
• Adjuvant radiation is used in certain cases to improve local control.

## 1 Introduction

Chondrosarcoma is a malignant tumor characterized with the formation of cartilage by tumor cells [1]. It appears to be the second leading sarcoma of bone after osteosarcoma, accounting for 10–20 % of all malignant bone tumor [2,3]. The most common sites of chondrosarcoma are pelvis, femur and ribs, but less common in head and neck. The involved sites in head and neck region are likely to be larynx, maxilla sinus, and skull base [4]. Chondrosarcoma of the temporo-mandibular joint (TMJ) is extremely rare, and to our best knowledge, they are only approximately 30 cases were reported worldwide [5].

Surgery is first choice in management strategy of chondrosarcoma, whereas radiation is used as an adjuvant therapy in selective cases. Besides, the role of chemotherapy is unclear [6]. Treatment of chondrosarcoma of the TMJ is still challenging because of essentially adjacent structures, for instance parotid gland, facial nerve, and skull base structures. Some complications like facial nerve paralysis, TMJ dysfunction can occur during treatment [5].

In this report, we present a case of chondrosarcoma in the right TMJ, which is treated successfully with surgery and radiation therapy. This work has been reported in line with the SCARE criteria [7].

## 2 Case presentation

**Fig. 1:**
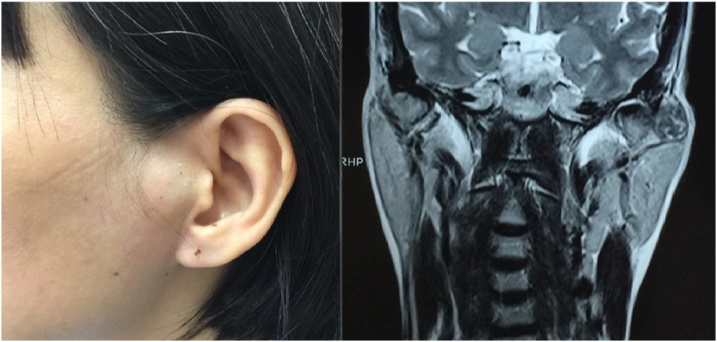
A mass in the left pre-auricular region in clinical exam and MRI.

A 47-year-old woman was admitted to our hospital complaining of a mass in her face for 2 months. She had mild pain in her left face when chewing, without any limitation in mouth opening movement. She did not experience any fever or weight loss. On examination, there was a 2-cm mass in the left pre-auricular region (Fig. 1). The lesion was hard, tender and covered by normal skin. No palpable cervical lymph nodes were found and no facial nerve paralysis was noticed.

Head and neck magnetic resonance imaging (MRI) showed a tumor in the left pre-auricular region with size of 16 × 19 mm, continuing with the left parotid gland. This tumor appeared as a mild decreased signal lesion in T2 and a mild increased signal lesion in T2 fatsat, comparing to the parotid gland. The tumor had clear and regular border, did not invade to surrounding tissue, and was associated with heterogeneous and widespread contrast enhancement. These characteristics suggested a parotid gland tumor (Fig. 1). A fine-needle aspiration of the mass was done with result of suspicious malignant cells.

**Fig. 2:**
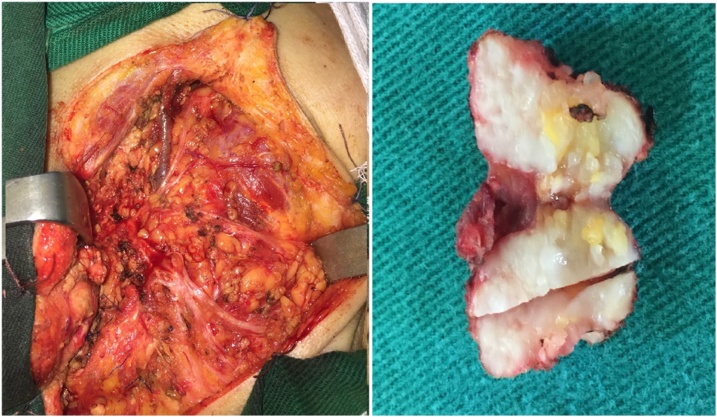
Intra-operative diagnosis was the TMJ chondrosarcoma.

**Fig. 3:**
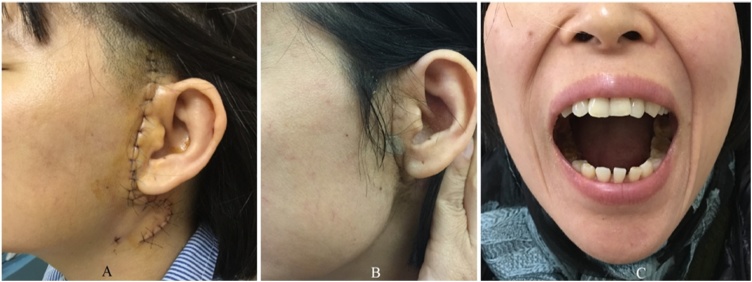
Patient 1 week after surgery (A) and 3 months after surgery (B and C).

Pre-operative diagnosis was suspicious left parotid gland cancer and the original treatment plan was total parotidectomy. In operation, after removing the superficial lobe of the left parotid gland, the lesion was found as a mass originating from the left TMJ with size of 2 × 2 cm, and without involvement in adjacent muscles or facial nerve (Fig. 2). As a result, intra-operative diagnosis was sarcoma of the left TMJ. Thus, tumor resection was performed with close margin. The final pathological result was low-grade chondrosarcoma, which was described as a hyaline cartilaginous proliferation, with stroma containing stellate, spindle-shaped and rounded cells, without mitosis and cellularity characteristics. Patient fully recovered after 1 month without any complications (Fig. 3).

Because the patient was evaluated as high risk of recurrence, she was indicated post-operative radiation therapy with total irradiated dose of 60 Gy in 30 fractions (5 days a week) with 3D conformal radiation technique. No severe radiation-related complications were reported during treatment. This patient was closely followed up afterwards. No adverse events were recorded and MRI performed a year after treatment showed no recurrence.

## 3 Discussion

Chondrosarcoma is a malignant tumor originated from cartilage cells, which is commonly seen in adults from 30 to 60 years of age and hardly occur in those under 20 years old [1]. It accounts for approximately 10–20% of bone malignancy, in which only 1%–12% cases are found in the head and neck region and the most common sites is larynx, maxilla and skull base [4,5]. Chondrosarcoma of the TMJ is rare [3,5,8,9]. The common symptoms of TMJ chondrosaroma are mass in pre-auricular area, spontaneous pain or pain when chewing, whereas trismus and laterodevitation at mouth opening are uncommon [8]. The duration between symptom onset and diagnosis ranges from 3 to 24 months, and might even be 6–8 years as reported in two cases [10]. The delayed diagnosis may be due to the facts that these symptoms are neither specific nor prominent and the disease usually progress slowly [9,11].

Panorama X-ray, maxillofacial computed tomography (CT) scan and MRI play essential roles in the diagnosis of the TMJ chondrosarcoma. Because X-ray normally provides inadequate information of the extension of the lesion, other imaging modalities such as maxillofacial CT or MRI should be carried out to confirm the diagnosis and decide treatment plan. The specific lesion characteristics are a non-enhancing mass with flocculent calcification at the level of the condyle affected, condylar deformity, with or without erosion of adjacent bone such as the skull base and the external auditory canal. In most cases, expansion of the articular space and rise in the length of condylar neck with radiopacity of the condyle are frequently observed [5,8,9,12].

Generally, fine needle aspiration is insufficient to distinguish the TMJ chondrosarcoma from the TMJ osteogenic sarcoma, parotid pleomorphic adenoma, parotid carcinoma and the TMJ chondroma. Therefore, open biopsy is important to reach the final diagnosis [8,9,12]. However, in a case reported by Sesenna et al., the initial diagnosis was still misdiagnosed of pleomorphic adenoma even based on both FNA and biopsy results [12]. In our case, the pre-operative diagnosis was the left parotid gland cancer because the result of FNA revealed suspicious malignant cells and maxillofacial MRI suggested a tumor continuing with the left parotid gland. In operation, we found that the lesion originated from the left TMJ, suggesting a sarcoma of the left TMJ. The final pathological result confirmed the diagnosis as low-grade chondrosarcoma. Chondrosarcoma of the TMJ area contains similar histopathological features to other regions. Microscopically, this tumor is characterized by a hyaline cartilaginous proliferation, with stroma containing stellate, spindle-shaped, or rounded cells [3]. Criteria for diagnosis include an increased number of cells, expansion of the nuclei, cell with binucleate forms or giant cell tumor formation [5,10]. The chondrosarcoma has been classified into grade I, II or III based on the frequency of mitosis, cellularity, and nucleus size [13]. However, there can be difficulty in distinguishing between a well-differentiated chondrosarcoma (Grade I) and a chondroma because of less cellularity and smaller cell [12].

The goals of treatment for patient with the TMJ chondrosarcoma include minimizing the risks of local recurrence, metastatic disease, and death from disease, whereas maintaining the function of the patients [5,6]. Normally, wide tumor resection with negative margins plays the most important role in management strategy, while cervical node dissection is unnecessary due to low rate of regional lymph node metastasis. In group of patients with low-grade, non-radiographically aggressive characteristics, for instance soft tissue invasion, adjacent bone destruction, and skull base invasion, intra-lesional procedure can be performed with no significant difference in overall survival, metastatic rate and local recurrence, comparing to wide resection [6]. In previous studies, the role of radiation therapy was demonstrated for salvage treatment in unresectable disease and adjuvant treatment in post-operative patient with inadequate surgical therapy [5,10]. The dose needed to control chondrosarcoma is at least 60 Gy with standard fraction from 1.8 to 2 Gy/day [6]. The role of chemotherapy in patients with local and advanced chondrosarcoma remains undefined. However, recent data have suggested a possible role in certain subtypes of chondrosarcoma, specifically the dedifferentiated and mesenchymal variants [6,14,15]. In our case, due to suspicious inadequate surgery, this patient was indicated post-operative radiation therapy with total irradiated dose of 60 Gy in 30 fractions (5 days a week) with 3D conformal technique. During treatment, no severe radiation-related complications were noticed. There was no sign of recurrence after follow-up for 6 months.

## 4 Conclusion

Chondrosarcoma of the TMJ is very rare. It need to distinguish from some diseases likely to TMJ osteogenic sarcoma, parotid pleomorphic adenoma, parotid gland carcinoma and TMJ chondroma. The most important treatment is wide tumor resection. Radiation therapy is useful in unresectable disease or adjuvant treatment in post-operative patients with inadequate surgical therapy.

## Declaration of Competing Interest

None.

## Funding

None.

## Ethical approval

The study was approved by our research committee, Hanoi Medical University Hospital, Hanoi, Vietnam and National Cancer Hospital, Hanoi, Vietnam.

## Consent

The publication of this study has been consented by patient.

## Author contribution

Quang V. Le: Professor, main surgeon.

Dang V. Nguyen: Radiation oncologist, treated the patient.

Hung V. Nguyen: Radiation oncologist, wrote manuscript.

Thanh D. Hoang: Assistant surgeon, wrote manuscript.

Duy Q. Ngo: Assistant surgeon, revised manuscript.

Tung T. Ngo: Professor, revised manuscript.

## Registration of research studies

This is not a first-in-human study, thus it is not needed.

## Guarantor

Quang V. Le, Professor, M.D, Ph.D.

## Provenance and peer review

Not commissioned, externally peer-reviewed.
